# Involving stakeholders in the design of ecological momentary assessment research: An example from smoking cessation

**DOI:** 10.1371/journal.pone.0217150

**Published:** 2019-05-22

**Authors:** Peter D. Soyster, Aaron J. Fisher

**Affiliations:** Department of Psychology, University of California, Berkeley, Berkeley, California, United States of America; The MetroHealth System and Case Western Reserve University, UNITED STATES

## Abstract

Ecological momentary assessment (EMA) is a data collection method that involves repeated sampling of participants’ real-time experience and behavior as they unfold in context. A primary challenge in EMA research is to design surveys that adequately assess constructs of interest while minimizing participant burden. To achieve this balance, researchers must make decisions regarding which constructs should be included and how those constructs should be assessed. To date, a dearth of direction exists for how to best design and carry out EMA studies. The lack of guidelines renders it difficult to systematically compare findings across EMA studies. Study design decisions may be improved by including input from potential research participants (stakeholders). The goal of the present paper is to introduce a general approach for including stakeholders in the development of EMA research design. Rather than suggesting rigid prescriptive guidelines (e.g., the *correct* number of survey items), we present a systematic and reproducible process through which extant research and stakeholder experience can be leveraged to make design decisions. To that end, we report methods and results for a series of focus group discussions with current tobacco users that were conducted to inform the design of an EMA study aimed at identifying person-specific mechanisms driving tobacco use. We conclude by providing recommendations for item-selection procedures in EMA studies.

## Introduction

Ecological momentary assessment (EMA) is a data collection method that involves repeated sampling of participants’ real-time experience and behavior as they unfold during daily life [1]. In contrast to other longitudinal data collection methods (e.g., longitudinal panel study), EMA typically results in many observations per participant over a relatively short period of time—often a few days to several weeks. Rather than assessing global retrospective self-reports, which can be limited by recall bias, EMA captures participants’ self-reported experiences and behaviors in real-time, in natural contexts. EMA studies routinely yield anywhere from dozens to hundreds of observations per participant (frequently referred to as *intensive longitudinal data* or *intensive repeated measures* [2]). The large number of observations per participant allows for the statistical examination of within-person processes as they unfold over time. EMA methods have been employed to study a wide range of behavioral and emotional phenomena, including emotional avoidance [3], substance use [4], and nutrition behaviors [5]. In practice, participants in EMA studies provide in-the-moment reports of their experience and behavior using a paper and pencil diary, responses to text messages, a smartphone app, or wearable device, as they go about their daily lives. These data can be quantitative (e.g., numerical ratings of mood) or qualitative (e.g., free-response text), and are collected actively through self-report or passively through mobile phone or wearable sensors (e.g., fitness tracking watch).

There are three sampling paradigms used in EMA studies; *event-contingent* (participants initiate a response when a predefined event has occurred), *signal-contingent* (participants are prompted at random times to get a representative sample of their experiences), and *time-contingent* (participants are prompted on a fixed time schedule) [6]. Many studies utilize more than one type of sampling process (e.g., signal-contingent sampling 4 times per day and participants initiate a report if an event of interest occurred between random surveys) [1,4]. Among published EMA studies, sampling frequency varies widely, from once per day, to as many as 60 times per day [7]. The sampling period–the duration of days or weeks that an individual participates in EMA–shows similar variability across studies.

In psychological and behavioral health research, EMA has traditionally been used to collect high-granularity data on within-person processes (e.g., moment to moment relationship between stress and alcohol craving for *Participant X*). These data are collapsed across participants to understand generalizable patterns of—or between-person differences in—these within-person processes (e.g., the average moment to moment relationship between stress and alcohol craving for *Population X*). In early applications of EMA design, intensive longitudinal data were often substantially reduced in granularity by averaging responses across days [8], or weeks [9]. Fortunately, as the number of researchers using EMA designs has increased [2], the statistical methods used for analyzing such data has become more sophisticated [10] allowing for more detailed analysis of within-person processes. Recently, EMA has begun to be used for person-specific (idiographic) research [11, 12]. Such approaches utilize EMA, and an accompanying suite of specialized statistical analyses (e.g., [13, 14]) to understand psychological phenomena as they exist within a specific person. In a person-specific approach, EMA surveys can measure a set of possible mechanisms leading to outcomes of interest (e.g., anxiety, substance use), and statistical analyses can identify which mechanisms appear to be relevant for a given person. These types of analyses can be completed pre-treatment and used to guide treatment selection and personalization [15].

Despite the variety of applications of EMA, currently there are not clear guidelines regarding how best to construct EMA studies. Some authors have characterized common elements of EMA studies [4] but often suggest a ‘common sense’ approach wherein prospective researchers use their intuition or best judgment to design surveys they think will best answer their research question. Thus, EMA surveys are highly variable in terms of the types of questions included, the number of assessments per day, and the length of the sampling period [16]. Flexibility in EMA design is desirable as it allows for researchers to collect data in a way that fits the goals of a given study; however, as EMA studies become increasingly common, methods are needed to guide best practices in designing assessment protocols that balance idiosyncrasy and flexibility, for meeting individual study and participant needs, with consistency and standardization, for comparison of results across studies.

The question of how best to select EMA survey items is an open and important one. For many outcomes and predictors of interest, extant theoretical and empirical work may provide a valuable resource from which to select potential constructs or items for intensive repeated measurement. However, for a variety of reasons–particularly participant burden–it is impractical to assess the complete set of possible variables. Large batteries of self-report measures are not practical and are unlikely to be tolerable for research participants under conditions of intensive repeated measurement [17]. Thus, there is an impetus for researchers to select a restricted set of items that provide adequate coverage for constructs that may be otherwise best-measured by larger batteries.

There are several techniques EMA researchers could employ to select items from larger validated retrospective measures. For instance, researchers could select a subset of items that have the highest factor loading on the construct of interest. While intuitive, this approach may not be optimal as it neglects that cross-sectional and in-the-moment (i.e. time-varying) measures are inherently different and, therefore, can yield substantially different results [18]. Estimates may fail to generalize across time, individual persons, or both [19]. Items drawn from a validated cross-sectional measure, but one that has not been shown to generalize across time, would likely not be useful in the context of EMA. Additionally, latent factor indicators are assumed to be conditionally independent and interchangeable, rather than representing unique sources of variance. Such a position is contrary to recent network theories of measurement [20].

Another approach is to generate a single item to assess each construct. As has been reported elsewhere [21], there is mixed evidence about the effectiveness of single-item measures in cross-sectional research. In some studies, single items have been shown to perform as well as multi-item scales for concrete constructs [22, 23]; others have concluded that multi-item measures are more valid [24]. In EMA research, participants often report on unidimensional constructs in terms of their current experience [16] (e.g., intensity of fatigue). In these cases, a single well-chosen item has been shown to be sufficient [21, 25].

Finally, researchers could simply rely on the precedent set by previous EMA studies. The recent proliferation of EMA studies has resulted in a large body of work from which researchers can select items. However, due to the large variability in study designs described above, there is unlikely to be a clearly superior choice. Additionally, while extant studies report the items used in the investigation, detailed explanation of the rationale for selecting included items is not routinely reported.

As researchers work to select a face-valid, parsimonious, and comprehensive set of items, difficult decisions must be made regarding which constructs should be included, and how those constructs should be assessed. We propose that engagement with potential research participants (*stakeholders*) will greatly inform, and strengthen these choices [26].

### Engaging stakeholders in research design

In 2008, the National Institute of Mental Health (NIMH) first published the Strategic Plan for Research, outlining four strategies to accelerate progress in both basic and clinical science [27]. Strategy 4.3 highlighted the need to involve stakeholders in all aspects of the research pipeline in order to increase the effectiveness of mental health interventions. Above and beyond the many benefits to including stakeholders in the research process [28], there are several benefits that are particularly relevant for EMA designs. Stakeholders can provide invaluable assistance to researchers in generating and narrowing down lists of items that could be included in EMA surveys to capture mechanisms related to the outcomes of interest. Additionally, stakeholders can provide important information–prior to expensive pilot testing–regarding the feasibility and acceptability of the proposed EMA assessment strategy within a given population.

Focus groups with stakeholders may be particularly helpful during the design phase of EMA research [29]. Focus groups are structured or semi-structured conversations conducted to gather stakeholder insights on shared or individual perspectives around specific topics. Advantages of focus groups include their ability to explore stakeholders’ individual knowledge and experiences, while simultaneously capitalizing on group dynamics and interpersonal exchanges among participants [30]. Thus, in addition to developing standardized methods for EMA survey construction, researchers should endeavor to include stakeholders in the early stages of design to improve the relevance and acceptability of EMA research.

The goal of the present paper is to introduce a general approach for including stakeholders in the development of EMA research design. To that end, we report methods and results from a series of focus group discussions with current tobacco users that were conducted to inform the design of an idiographic study aimed at identifying person-specific mechanisms driving tobacco use. We conclude by providing recommendations, based on our experiences, for item-selection procedures in future EMA studies.

### An example from smoking cessation

Tobacco use is the leading cause of preventable death in the United States, resulting in an estimated 480,000 deaths per year [31]. Beyond early mortality, the consequences of tobacco use are wide-ranging, extending to physical health, mental health, and even the ability to find employment–particularly for those from vulnerable populations (e.g., those with mental illness, LGBTQ+) [31–34]. In light of the mortality and impairment associated with tobacco use, reducing the number of people who smoke is a major goal in behavioral and public health. There is a dire need for effective and affordable tobacco treatments (TT) to reduce use.

Decades of tobacco research have yielded insights into what mechanisms should be targeted in evidence-based TT [34]. Although many interventions have demonstrated effectiveness in clinical trials, the majority of people who receive TT fail to quit [35]. One potential area for improving TT outcomes is personalization: modifying the content and presentation of interventions to make them more relevant for a given person or population. Existing research is mixed regarding the effects of specific mechanisms on tobacco use (e.g., the relation between negative affect and subsequent tobacco use). As a result, there has recently been a call for novel assessment approaches that would yield person-specific information about mechanisms driving tobacco use, which would allow for the personalization of TT [36, 37].

Given that existing research is equivocal regarding which variables are most related to subsequent tobacco use, the present study utilized an approach to examine the experiences of stakeholders regarding what they believe drives their momentary choices to smoke or not. Specifically, we aimed to (1) review existing research in order to develop a list of constructs that have been empirically or theoretically linked to tobacco use; (2) examine current tobacco users’ thoughts and attitudes about the relevance of these constructs to their own smoking behavior; and (3) synthesize these information sources to develop survey items to be used in a future EMA study of tobacco use.

## Methods

All study procedures were approved by the University of California, Berkeley Committee for Protection of Human Subjects and all participants provided informed consent prior to participation.

### Aim 1

To address the first aim, we sought to compile a relatively short list of constructs for potential inclusion in a future EMA study of tobacco use, to be refined through the second and third aims. Specifically, we were interested in mechanisms relating to increased or decreased likelihood of current tobacco users choosing to smoke at a given point in time—rather than mechanisms related to the initiation of smoking in non-users. As the ultimate goal of the future EMA study was to identify personalized smoking cessation treatment targets, we were specifically interested in identifying constructs that (1) had a high probability of being relevant to current tobacco users, (2) would be likely to vary in level or intensity within individuals over the course of a day, and (3) were potentially modifiable through a known evidence-based intervention. We also aimed to understand the typical wording, response format, and sampling frequency used to measure these constructs in other published, peer-reviewed EMA studies of tobacco use.

To those ends, we conducted a systematized review [38] of literature regarding within-person mechanisms relating to tobacco use. Due to the applied nature of this work, we elected to forgo a formal systematic review, and instead conducted a comprehensive review of previously published studies without incorporating strict metrics for analyzing the quality of each study.

We began by utilizing a social cognitive theory [39] framework to guide our search for constructs related to health behaviors in general, expanding our search to include studies of tobacco-specific constructs. A trained research assistant searched the Google Scholar, PsychINFO, and PubMed databases to identify studies for possible inclusion. Search terms and phrases were combined and reflected a focus on tobacco use (*tobacco*, *smoking*, *cigarette*, and *nicotine*), the employed methodology (*ecological momentary assessment* and *experience sampling methods*), and constructs included in the EMA surveys (*within-individual*, *mechanisms*, *social cognitive theory*, *self-efficacy*, *locus of control*, *expectancy*, *health beliefs*, *craving*, *withdrawal*, *motivation to quit*, *stages of change*, *negative affect*, *positive affect*, *stress*, and *impulsivity*). No restriction was placed on the year of publication. Searches were restricted to English language, peer-reviewed studies. Databases were searched in May of 2017. For each combination of search terms, all indexed article titles were reviewed, and potentially relevant articles (i.e. those relating to within-person processes driving tobacco use) were retained.

After de-duplication, these search procedures resulted in an initial list of 279 unique articles. Two researchers then independently reviewed the abstracts for these articles and manually excluded papers that (1) did not include a focus on tobacco use, (2) did not include any application of EMA methods (reviews of EMA studies were retained), (3) only utilized EMA during or after a quit-smoking attempt, or (4) were secondary analyses of data presented in an already included study. Interrater reliability was high among the reviewers (92% agreement) and all disagreements were discussed and settled before thematic analysis. In total, 52 studies were retained.

#### Thematic analysis

After identification, a trained research assistant reviewed the full text of each study to extract all reported variables included in the EMA surveys, the wording and response format for each item, item sampling rate, and the length of the sampling period (Table 1). The first author reviewed a random sample of 10 articles to confirm the validity of data extracted by the research assistant. No conflicting variable extraction was evidenced during this checking step.

**Table 1:** Frequencies of reviewed study characteristics.

Signal type	Frequency
Event-contingent	6
Signal-contingent	13
Time-contingent	4
Mixed	23
Could not be determined	0
Sampling frequency*	
1/day	1
3/day	3
4/day	9
5/day	3
6/day	2
7/day	1
8/day	1
9/day	2
Mixed	11
Could not be determined	2
**Sampling period**	
1 day	1
4 days	1
6 days	1
7 days	13
8 days	2
10 days	2
14 days	11
16 days	4
21 days	4
28 days	2
≥ 30 days	1
Mixed	4
Could not be determined	0
**Response format**	
Continuous numerical	5
Free response	1
Categorical	1
Likert type scale	15
Mixed	22
Could not be determined	2

This table excludes review studies; Mixed = a combination of the categories was used.

*Sampling frequency only represents the frequency of surveys initiated by the study (i.e., signal- and time-contingent surveys)

The authors then collaboratively thematically analyzed and coded the variables to reduce the data into meaningful discrete construct categories. There was a large degree of overlap in the definitions, and component parts, of purportedly distinct constructs in psychology [40]. For instance, having difficulty concentrating was found to be a shared indicator for depressive episodes, nicotine withdrawal, and impulsivity [41, 42]. To validly match potential constructs to specific evidence-based interventions (a criterion for construct inclusion in the present study), it was important to thematically analyze and classify the constructs used in previous studies in a way that resulted in minimal construct overlap. Initial construct categories were based on the descriptions provided in the reviewed studies (i.e., what the authors stated they were attempting to measure). This classification was done deductively; we utilized our knowledge of relevant psychological theory (e.g., *happy*, *positive*, and *enthusiastic* were coded as *positive emotions* [43]) to determine construct classification. As these construct categories were identified collaboratively between the two authors, and no new constructs were proposed (i.e., we did not attempt to codify a previously unknown construct), thus we did not formally assess trustworthiness or reliability.

From these constructs, we then generated a list of potential survey items—at least one for each construct—to be considered for inclusion in the final study (Table 2). In the case that there was majority agreement in how a construct was assessed among the reviewed studies, the most prominent item wording was used. In the case that there was no consensus among the reviewed studies, the wording of individual items was either pulled directly from an established measure of the construct (e.g., nicotine withdrawal [42]), or a new item was generated by the authors.

**Table 2:** EMA survey items presented to focus groups and final items chosen for study inclusion.

Construct	Items presented to focus groups	Percentage of participants who indicated item was relevant				Final items for each included construct
		Total (*N* = 19)	Com 1 (*N* = 7)	Com 2 (*N* = 4)	College (*N* = 8)	
**Craving / smoking urge**	• I want to smoke	68%	100%	25%	63%	• How strong is your urge/craving to smoke right now?
	• I am experiencing craving to smoke	74%	86%	50%	75%	
**Smoking cues**	• I am encountering smoking triggers	79%	86%	75%	75%	• People or situations are triggering me to smoke
	• People or situations are making me want to smoke	84%	100%	50%	88%	
**Social context**	• I am inspired by others not to smoke	63%	57%	50%	75%	• I am enjoying my interactions with other people • I feel comfortable in my current location/situation
**Nicotine withdrawal**	• I feel down or depressed	47%	43%	25%	63%	• I feel down or depressed
	• I feel anxious	84%	71%	100%	88%	• I feel stressed
	• I feel angry or frustrated	58%	71%	75%	38%	• I feel angry
	• I feel hungry	42%	57%	50%	25%	• I feel hungry
	• I am having difficulty concentrating	63%	57%	50%	75%	• I am having difficulty concentrating
**Smoking enjoyment**						• I enjoyed my last cigarette
**Positive emotions**	• I feel optimistic	47%	71%	25%	38%	• I feel happy
	• I feel positive	68%	100%	50%	38%	• I feel enthusiastic
	• I feel energetic	53%	42%	0%	38%	• I feel calm/relaxed
**Negative emotions**	• I feel pessimistic	42%	43%	25%	50%	• I feel irritable
	• I feel bored	84%	86%	50%	100%	• I feel bored
	• I feel stressed	74%	57%	75%	88%	• I feel frustrated
**Physiological arousal**	• I feel jittery	58%	86%	25%	50%	• I feel nervous/tense • I feel restless
**Bodily sensations**						• I feel tired
**Smoking valuations**	• I value the physical benefits of not smoking	79%	71%	100%	75%	
	• I value the mental and emotional benefits of not smoking	79%	71%	100%	75%	
	• I value the social benefits of not smoking	74%	71%	75%	75%	
**Motivation/ intention to quit**	• Not smoking is important to me	79%	57%	75%	100%	• I am motivated to quit smoking
	• I am motivated to not smoke	79%	57%	75%	100%	• I want to quit smoking
	• I intend to not smoke	79%	57%	100%	88%	
	• I want to quit smoking	84%	57%	100%	100%	
**Smoking expectancies**	• If I smoke, it will relax me	95%	100%	75%	100%	• A cigarette would improve my mood or make me feel better
	• If I smoke, I will feel better physically	42%	57%	75%	13%	
	• If I smoke, I will feel better mentally or emotionally	68%	71%	75%	63%	
**Smoking stigma**	• Smoking makes me feel bad about myself	53%	43%	50%	63%	• I am embarrassed/ashamed that I am a smoker
**Locus of control**	• The amount I smoke is within my own control	79%	100%	75%	63%	• The amount I smoke is within my own control
	• There are barriers or obstacles to reducing my smoking	74%	71%	100%	63%	
**Self-efficacy**	• I am capable of not smoking	58%	57%	50%	63%	• If I tried to quit smoking right now, I would be successful
	• Not smoking is difficult	79%	71%	100%	75%	
	• I feel confident that I will not smoke	32%	29%	75%	25%	
**Health beliefs**	• My smoking is hurting my health	95%	86%	100%	100%	• My smoking is hurting my health • My health would improve if I quit smoking
**Impulsivity**	• I can delay gratification	42%	57%	25%	38%	• I can delay gratification • I feel impulsive
	• I have self-control	58%	57%	25%	75%
	• My thoughts and decisions are rational and deliberate	74%	86%	25%	88%
	• My thoughts and decisions are driven by my emotions	74%	100%	50%	63%
	• My choices and behavior are impulsive	47%	86%	25%	25%

Com 1 = community member focus group #1; Com 2 = community focus group #2; College = undergraduate member focus group.

Finally, the sampling paradigm (e.g., event-, signal-, or time-contingent), sampling frequency, response format, and length of sampling period were reviewed, and the frequency of each approach was tabulated. When relevant, we weighted our proposed design choices to be consistent with the more frequently used design features. However, as the future EMA study intended to employ idiographic time series analyses, a relatively large number (>100) of roughly evenly spaced observations was required for each participant. This meant that in some cases, frequently used design methods (e.g., event-contingent sampling, only assessing constructs once per day) were deemed to be incompatible with the goals of the future EMA study.

### Aim 2

To address our second aim of understanding tobacco users’ attitudes about constructs relevant to their smoking behavior, we conducted three focus groups with current tobacco users to examine their thoughts and attitudes about the relevance of the items generated through Aim 1 to their own smoking behavior. Additionally, we sought participants’ input regarding additional constructs/items not identified through Aim 1. One group included participants from an undergraduate research participation pool, and two groups were composed of participants recruited from the larger San Francisco Bay Area community.

#### Participants

Participants (*N* = 19) were adults (*Mean age* = 33.6, *SD* = 14.5, range = 19–60) who self-identified as current tobacco users. Participants were drawn from communities from which we intended to recruit participants for a future study of person-specific factors driving tobacco use. Across the three focus groups, 11 participants (57%) identified as female, 8 identified as male. The groups were diverse with respect to race/ethnicity (32% white, 26% Black or African American, 26% Asian/Pacific Islander, 16% mixed or ‘other’) and sexual orientation (68% heterosexual, 5% homosexual, 16% bisexual/queer, 11% unsure or prefer not to disclose). Participants smoked an average of 7.6 cigarettes per day (*SD* = 6.61, range = 1–27) for an average of 16.2 years (*SD* = 16.2, range = 1–52). Eighty-four percent indicated that they were seriously thinking about quitting smoking in the next six months, with 47% indicating they were thinking about quitting in the next 30 days.

#### Procedure

Potential participants were recruited from advertisements on Craigslist, flyers posted in the community, and through an undergraduate research participation pool. Interested individuals were directed to an online screening survey designed to confirm eligibility for study inclusion. To be included in the study, participants were required to be adults with English-language proficiency, to report having smoked 100 or more cigarettes in their lifetime [44], and to report currently smoking ≥ 1 cigarette per week. Neither desire nor motivation to quit smoking were required to participate.

Eligible participants were invited to our lab at the University of California, Berkeley for enrollment and participation in a focus group. After completing consent procedures, participants’ self-reported smoking status was biochemically verified using exhaled carbon monoxide (*M* = 11.1ppm, *SD* = 7.8 ppm, range 2 ppm– 30 ppm) [45]. Before the focus groups began, participants completed a computer-based survey, which assessed participant demographics, current and past tobacco use, and a variety of psychological and emotional variables that have been linked to differential patterns of tobacco use (e.g., personality facets were assessed using the NEO Five Factor Inventory [46]; past-week anxiety was assessed using the Overall Anxiety Severity and Impairment Scale [47]). In addition to providing descriptive information about the sample of focus group participants, these measures were included to understand the length of time participants required to complete the baseline battery of measures we planned to include in the future EMA study. We recruited 7 participants for the undergraduate focus group, and a total of 12 participants for the two community member focus groups. Participants who completed the focus group were reimbursed with $25 or partial course credit.

Focus groups were conducted between July and August of 2017 and had an average duration of 60 minutes. The groups were facilitated by one of two graduate student researchers with training and experience in conducting qualitative research (F1: white, male, PhD student; F2: white, female, PhD student), with assistance from two research assistants. At the beginning of the focus groups, the facilitator explained how EMA research is conducted, and outlined the goals of the planned tobacco EMA study with the following statement:

*“The goal of this conversation is to share a list of factors that we think might cause people to smoke more or less throughout the day*. *We hope that you can tell us if you agree or disagree with our list, and let us know about any factors you think are missing from our list. We also hope that through this discussion you can share your experiences with smoking so that we make our final list of daily survey questions as relevant as possible”.*

Participants were provided a written list of 39 potential EMA items and were asked to indicate whether or not they thought each item related to their smoking (0 = *not related*, 1 = *related*). The group then discussed their reasons for marking items as related or unrelated to their smoking. For any item marked as related to smoking, participants could indicate if that item was particularly strongly related to their smoking (i.e., the item is *very related*). Participants were encouraged to focus their responses as to whether or not an item applied specifically to them, not whether they believed it may be important for others. After this initial discussion, facilitators used a semi-structured interview guide to ask open-ended questions, covering (1) likes and dislikes about smoking, (2) causes of smoking/refraining from smoking, (3) thoughts and feelings that occur immediately before and after smoking, (4) experiences of cigarette craving, and (5) experiences with past quit-attempts.

#### Directed content analysis

The focus groups were audio-recorded. One research assistant transcribed the audio recordings verbatim, and another checked the transcriptions for accuracy. After transcription, the group facilitators and two research assistants independently analyzed the transcripts using directed content analysis [48]. The goal of directed content analysis is to analyze new data (e.g., focus group transcripts) to validate or extend an extant theory. The present study did not specifically seek to validate the existence of the constructs identified through the first aim, as this work had already been completed by the reviewed studies. Instead, we sought to validate the assumption that each of proposed items would be relevant for some focus group participants but not others. While we did not have specific hypotheses regarding which items would be relevant to which participants, we used a deductive approach to extend our understanding of which constructs where relevant for the largest proportion of participants (through tabulating frequencies for the presence of a code), and to understand more about the function of each construct in relation to smoking. The initial list of codes consisted of the constructs identified through Aim 1 and were used to classify the content of participant statements. These constructs were compared among the coders, and through an iterative process, were used to extract sub-themes relevant to increased or decreased smoking. After determining the final list of construct codes, two research assistants coded the transcripts and tabulated frequencies for the presence of each code (Table 1). Intercoder agreement was high across each of the three transcripts (community 1 = 94%; community 2 = 92%, college = 95%). All coding disagreements were reviewed among the coding research assistants and first author, and a consensus vote was used to make a final coding determination.

Given the process-focused nature of EMA research, the coding team utilized a functional analysis approach [49] to identify constructs that were (1) antecedents of smoking behavior choices, (2) the behavior prompted by those antecedents (i.e. smoking or not smoking), and (3) the consequences of choosing whether or not to smoke. Put another way, this content analysis was not intended to comment on or fine-tune the definition of a given construct (e.g., *craving*); instead, we assessed whether participants felt craving was relevant to their smoking. This was the principal interest—to identify the most common and influential predictors of smoking. Additionally, we sought to identify potential mechanistic pathways through which craving led to increased or decreased smoking, and the consequences of those actions (i.e. the antecedents and consequences of craving).

### Aim 3

To synthesize the data collected through the previous aims, our research group held several meetings to review the data. Through these discussions, we developed a list of EMA survey items, integrating feedback from stakeholders and literature review. The goal of these discussions was to determine which constructs should be included (e.g., *negative emotions*) and the number and wording of survey items used to measure those constructs (e.g., *I feel sad)*.

#### Construct and item selection

**Fig 1:**
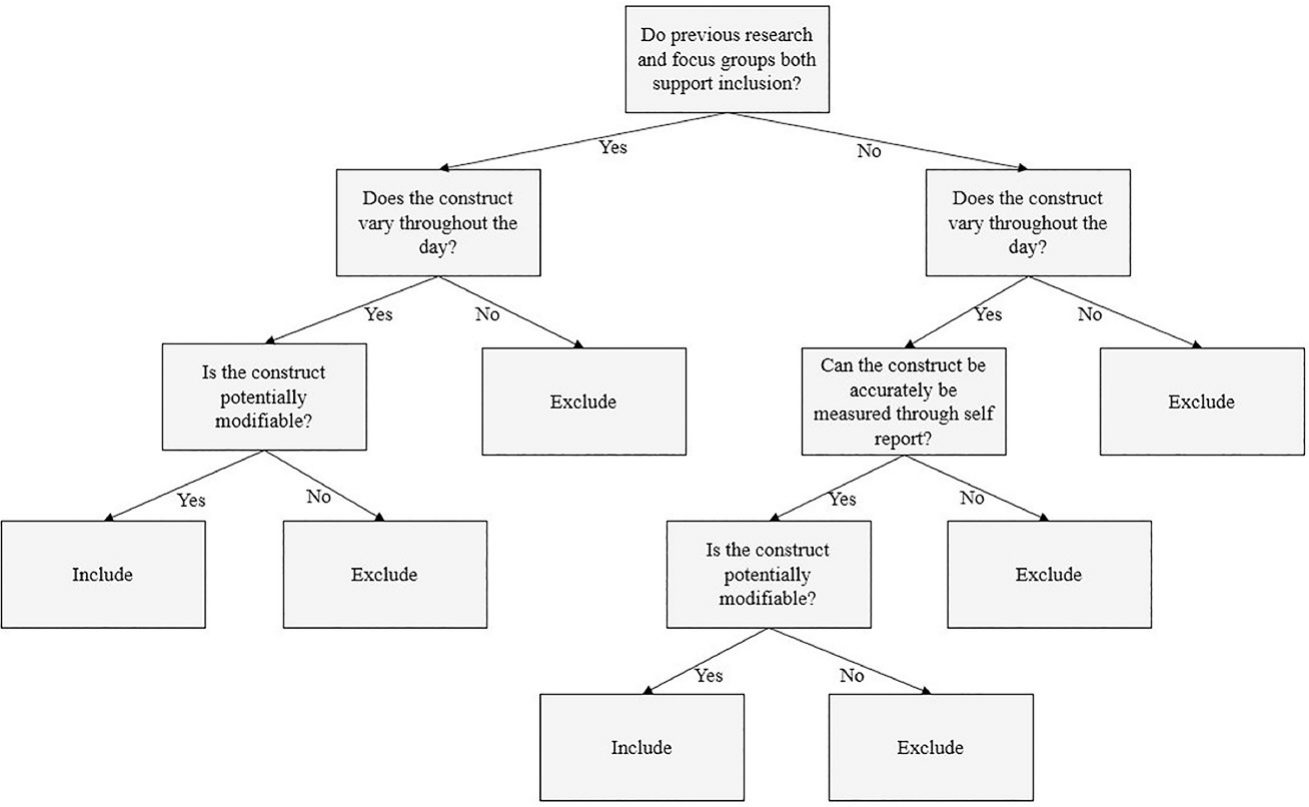
Decision rules used to determine construct inclusion. Conceptual diagram of the decision rules used to determine construct inclusion.

**Fig 2:**
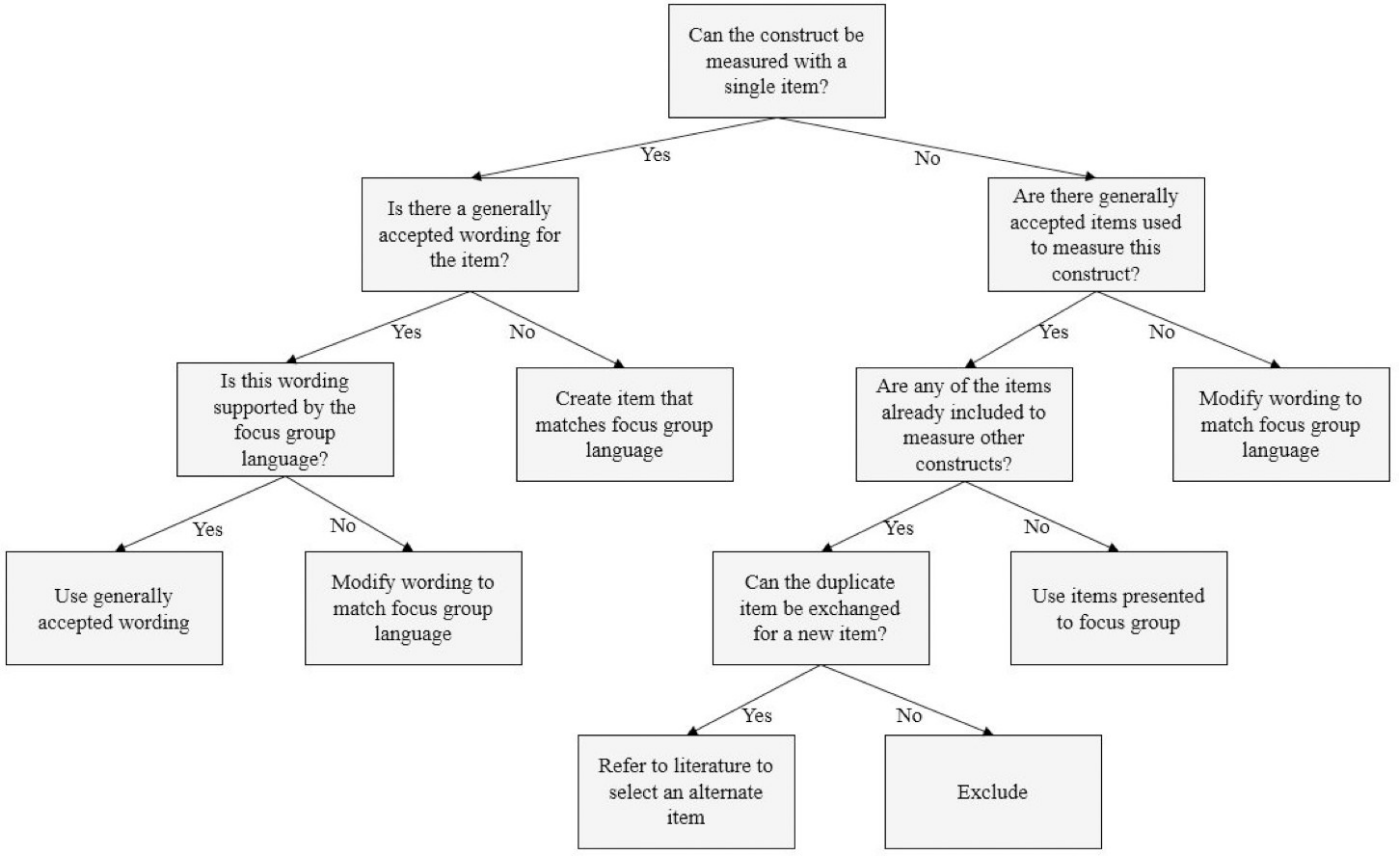
Decision rules used to determine survey item inclusion. Conceptual diagram of the decision rules used to determine survey item inclusion.

We started by defining a set of decision rules for determining which constructs should be assessed in the EMA survey (Fig 1). These decision rules were designed to reduce the list of constructs identified through the previous aims to those that (1) had a high probability of being relevant to current tobacco users, (2) would be likely to vary in level or intensity within individuals over the course of a day, and (3) were potentially modifiable through a known evidence-based intervention. After a list of constructs was codified, a similar process (Fig 2) was used to determine the number of items needed to measure each construct, and the specific wording of each item. In the case that our predetermined decision rules were insufficient to determine inclusion/exclusion status, a ruling was made by consensus vote. Similarly, if two items measuring the same construct had equal support for inclusion, consensus vote was used to select one item for inclusion.

## Results

### Aim 1

The reviewed studies exhibited substantial variability in the sampling paradigm (e.g., *event-*, *signal-*, or *time-contingent*), sampling frequency, response format, and length of sampling period (Table 1). Of note, 65% of the reviewed articles did not include sufficiently detailed method sections to extract all design-related details. The most frequently omitted detail was the complete wording used to assess all items. Based on the frequency characteristics of the reviewed studies, as well as the data requirements of idiographic time-series analyses, items were designed to be assessed 4 times per day on a *signal-contingent* schedule, for 30 days using a visual analog slider response scale ranging from 0 (*not at all*) to 100 (*as much as possible*).

We identified 12 construct categories from the EMA literature that were either theoretically or empirically linked to variations in smoking behavior (Table 2). Three additional constructs (*impulsivity*, *smoking valuations*, and *health beliefs about smoking*) were also included. While these constructs were not assessed in the EMA surveys of the reviewed studies, they are frequently included in theoretical models of smoking behavior (e.g., [39, 50]) and as covariates in cross-sectional studies of smoking [51]. Additionally, they fulfilled the criteria outlined for potential inclusion (i.e., a within-person process that could be modified by a known evidence-based intervention). From these constructs, we developed 39 EMA survey items to be considered by the focus groups.

For some emotion items, previous studies had categorized the same item (e.g., *I feel down or depressed*) as indicating either an affective valence (e.g., *negative emotions*) or a component of nicotine withdrawal. In these cases, we used a validated measure of nicotine withdrawal (Minnesota Nicotine Withdrawal Scale) [42] to select the emotion items that would represent components of nicotine withdrawal. We then developed non-withdrawal-related emotion items to indicate high- and low-activation positive and negative emotions [52].

### Aim 2

Individual participant ratings of the relevance of specific EMA items, as well as deidentified focus group transcripts can be accessed through the Open Science Framework website at: https://osf.io/wch9m/. A summary of participant ratings of the relevance of specific EMA items to their smoking choices are presented in Table 2. *Anxious*, *bored*, *smoking will relax me*, *smoking is hurting my health*, *people or situations are making me want to smoke*, and *I want to quit smoking* were items most frequently endorsed as related to smoking behavior. The least frequently endorsed items were *pessimistic*, *hungry*, *feel able to delay gratification*, *confident I will not smoke*, *and smoking will make me feel better physically*.

The focus group transcripts were coded to identify participants’ self-reported antecedents of smoking choices, and the consequences of those choices. The coding process yielded 7 classes of antecedents related to increased smoking (emotions, bodily sensations, thoughts, actions, location, social influences, and habit) and 6 such classes related to decreased smoking (emotions, bodily sensations, thoughts, actions, location, and social influences). Consequences of smoking behavior were broken down into *internal* (within-person) and *external* (interpersonal or environmental) consequences. These categories were further broken down to represent whether the participant experienced the consequence as positive or negative (Table 3).

**Table 3:** Antecedents and consequences of smoking behavior.

Antecedents	Behavior	Consequences	
**Emotions**: Sad, angry, anxious, bored, irritable, impulsive, wronged, positive, happy, energetic, short-tempered, lonely **Bodily sensations**: Craving, heart beating fast, tired, hungry, headache, cloudy-headed, shaky hands, tension **Thoughts:** I want to reward myself, I need to calm my nerves, imagine smoking a cigarette, I need to get away from this situation, there is no way to solve this problem, I don't care about quitting, I don't smoke that much **Actions:** Driving, talking on phone, drinking coffee, drinking alcohol, working, eating, exercise, waiting for an appointment **Location:** Car, work, walking outside, at a party **Social:** Being around others who are smoking, making small talk with someone I don't know well, being around a large group of people **Habit:** Not aware of my smoking- I just do it, first thing in the morning, smoke at same time every day	**Increased smoking**	**Internal**	
		**Positive**	**Negative**
		• Feel better physically and emotionally • Relaxing/ relieves stress • Time to think/ reflect • Enhances other positive experiences (esp. other drug use) • Reduces hunger • Pleasure from the act of smoking • Increases motivation	• Increased heartrate • Shame, embarrassed, guilt • Self-conscious about smell • Relief from stress is very short, then anxiety returns • Concerns about health • Harder to breathe
		**External**	
		**Positive**	**Negative**
		• Fit in socially • Break from work • Form community with other smokers • Get to go outside	• Smell bad on hands and clothes • Unattractive to others • Other smokers are drawn to you • Judgement from others
**Emotions:** Interested in work, positive, proud, energetic **Bodily sensations**: Hungry, nauseated/ dizzy **Thoughts:** I want to quit smoking, value benefits of not smoking, embarrassed of being a smoker, I haven't earned a cigarette yet, thinking about health consequences of smoking, I can't afford cigarettes **Actions:** Chewing gum, drinking water, using nicotine replacement product, telling people you are not going to smoke, meditating **Location:** Not going to bar, staying at home, home of non-smoking friend **Social:** Being around friends/family who don't smoke, want to set a good example for kids, seeing older people still smoke while they are sick	**Decreased smoking**	**Internal**	
		**Positive**	**Negative**
		• Reduces concern about smoke smell • Feel better about health	• Increased appetite • Irritable/ frustrated • Headache • Feel awkward in social situations • Muscle tension • Difficulty concentrating
		**External**	
		**Positive**	**Negative**
		• Look more professional at work • Friends/family are proud • Use inhaler less/ cough less	• Fewer breaks at work • Miss conversations with friends

The antecedent and consequence classes were largely consistent across focus groups. Of note, the undergraduate focus group reported fewer experiences related to nicotine withdrawal compared to the other groups. Further, while all groups reported that negative interpersonal interactions led to increased smoking, the specific emotion characterizing those interactions as negative differed between the undergraduates and the community members. Undergraduate focus group members tended to smoke more in response to *awkward* interpersonal interactions (e.g., making small talk with someone they didn’t know well), while the community focus group members tended to smoke more to deal with *frustrating* interpersonal interactions (e.g., avoiding an argument with a boss or family member).

### Aim 3

Figs 1 and 2 present conceptual diagrams of the decision rules used to determine inclusion/exclusion for constructs and items, respectively. This process resulted in a total of 30 items assessing the following constructs: cigarettes smoked since the previous survey, craving, nicotine withdrawal, smoking cues, health beliefs about smoking, smoking self-efficacy, locus of control for smoking, smoking expectancies, motivation to quit smoking, internalized smoking stigma, smoking enjoyment, positive emotions, negative emotions, physiological arousal, social context, impulsivity, and bodily sensations. We added four additional items to be completed only at the first survey of the day: three items measuring sleep quality for the previous night and one item asking the participant to predict the number of cigarettes they expected to smoke that day. Due to their correlation with several items in the EMA surveys (e.g., *fatigue*, *restlessness*, *I want to quit smoking*), these items were included so that they could be used as day-level covariates in the planned statistical analyses of the EMA data. Two constructs identified through literature review, cognitions and smoking valuations, were excluded through consensus vote as they were determined to be higher order constructs that encompassed one or more already included constructs (“cognitions” could include craving, internalized smoking stigma, health beliefs about smoking, and smoking valuations; “smoking valuations” could include smoking expectancies, smoking enjoyment)

## Discussion

A primary challenge in EMA research is to design surveys that adequately assess constructs of interest while minimizing participant burden. To achieve this balance, researchers must make difficult decisions about which constructs should be included, and how those constructs should be assessed [16]. Although flexibility in research design is important, each of these decisions carries with it the possibility of introducing researcher bias and other forms of imprecision that can threaten the generalizability of the study methods and results. Item selection can be even more challenging for person-specific EMA studies, as the *most relevant* constructs may vary substantially from person to person. Thus, it is critical to move toward the development of best practice guidelines that can guide selection and design decisions for varying EMA methodologies and research designs.

In the present study we outlined a series of steps researchers can take to improve the reproducibility, face validity, and potential relevance of EMA studies. The approach we suggest does not completely remove the potential for researcher bias or other sources of imprecision, but rather provides a framework for making design decisions that incorporates input from multiple sources of data, including extant theories, empirical data, and stakeholder perspectives. In practice, the exact rules used to make design decisions should depend on the goals of the study, but we have attempted to outline a transferable and replicable process through which these decisions can be made.

The first step addresses the importance of utilizing past research to inform study design and item selection. Reviewing the methods and results of previous studies is a nearly-universal step in the design of a new study, but this process can present specific challenges in the context of EMA research. In many cases, extant measures for constructs of interest will be too lengthy to administer multiple times per day. Further, often there exist multiple validated measures of the same construct. In cross-sectional research, researchers often opt to include multiple measurements of the same construct, yet this practice is likely impractical in the context of EMA studies. Additionally, many validated measures have been designed to assess participants’ global or trait-like levels of a construct (e.g., the average intensity of depressed mood), insensitive to all but the most macroscopic variations. However, EMA studies are often interested in fluctuations in level from moment to moment or day to day. Phraseologies need to be employed that can assess real-time, momentary variation. Furthermore, as was evidenced by the present review, previous research may omit important details—such as the exact wording of EMA items—making it difficult to precisely replicate past work. Any modification to the wording of a validated scale should be done with caution, as it is unclear how such changes may affect the measurement properties of that scale. The framework we suggest treats literature review as a hypothesis-generating step through which researchers can lean on existing empirical knowledge to generate lists of potentially relevant constructs or items. We find that this approach strikes a favorable balance between (1) relying too heavily on existing methods that may not be well-suited for EMA research, and (2) the ‘start from scratch’ approach.

The second step allows for the hypotheses generated through literature review to be presented to stakeholders for comment and refinement. Stakeholder involvement in research design has been recommended by funding bodies (c.f. [27]), and provides valuable input regarding whether proposed constructs are likely to be relevant to the study’s populations of interest and their lived experiences. Beyond the decision of *which* constructs to measure, it is crucial that researchers use the same language stakeholders use to describe constructs [16]. Direct conversations with stakeholders are a low-cost way for researchers to achieve this goal. We argue that if the language used by stakeholders to describe their subjective experience doesn’t match the wording of potential items capturing that construct—even if the construct has received strong empirical support, or academically-interests researchers—the item should be modified or dropped from the survey. This was the case in the tobacco example presented above, where we opted to change the item *I feel anxious* to *I feel stressed* after the results of the focus groups revealed that the research team’s formal definition of anxiety (i.e. apprehensive unease, distress) matched what participants described as *stressed*. Without this knowledge, any inferences we attempted to draw from the relations between smoking and anxiety would probably be compromised.

Importantly, this process can also identify constructs or items that previous literature may have missed. Having a rich body of literature to pull from can be tremendously helpful when designing a new study, but it may also serve to constrain researchers’ ability to critically consider novel areas of inquiry. Listening to the perspectives of stakeholders allows for researchers, who are experts in the academic understanding of a behavior or disorder, to learn from stakeholders, who are experts in the lived experience of a behavior or disorder. Because psychological and medical research frequently suffers from lack of diverse participant populations [53], it is likely that direct conversations with diverse stakeholders will lead to insights not available through literature review alone.

Finally, we propose synthesizing literature review, stakeholder input, and research needs as a way to create maximally effective EMA study designs. In an ideal situation, data gathered through literature review would be largely concordant with the researchers’ assumptions and stakeholder perspectives, in which case this final step would merely involve confirming the agreement among the data sources. We expect, and our experience has shown, that this ideal situation is unlikely to be the case. Instead, we advise researchers to expect some degree of conflicting or non-overlapping conclusions during qualitative data collection, and to preemptively codify decision rules for handling such variability. In the tobacco use study presented here, these rules were designed to satisfy the following conditions: (1) Any included construct should operate within the person and should be theoretically modifiable through an existing evidence-based intervention; (2) constructs should be measured with the smallest number of items that could feasibly capture variability in that construct; (3) the wording of items should, whenever possible, favor the language used by stakeholders; and (4) in the case that these conditions are insufficient to make a decision, a final determination will be reached through consensus vote among the researchers. These conditions, and the specific decision rules that are derived from them, will vary among studies but we believe the process of creating such rules is transferable to other investigations.

In some ways, the process we have suggested does not greatly differ from the study design procedures many researchers already use [4]. Indeed, it could be argued that we have simply outlined formal steps through which the ‘common sense’ approach can operate. For example, this process would not prevent researchers from consulting with stakeholders and then completely disregarding their insights, or from cherry-picking the evidence to support their a priori preferences. It is true that the methods proposed in this paper do not remove the ability of researchers to use their judgement to make final design decisions. To do so, we believe, would be overly restrictive and would limit the potential for innovation in study methods. Instead, we seek to empower researchers to make evidence-based judgements by engaging in steps that increase the pool of relevant data researchers have at their disposal to make decisions.

The results of the present work should be interpreted in the light of several limitations. Whereas we have presented the steps we undertook to design a person-specific EMA study of tobacco use, there was no application of the EMA survey in the present work. Pending the outcomes of that study, as well as the results future investigations utilizing the proposed methodology, the procedure outlined in this paper will require additional methodology for refinement in practice. Additionally, as was reported in the methods section, practical constraints (e.g., limited budget and time for preliminary study design) reduced our ability to fully implement some steps. For example, for Aim 1, we conducted a systematized, rather than systematic review of the literature [38]. While this review methodology incorporates aspects of a systematic review (e.g., comprehensive and replicable searching procedures), it is often reduced in scope and can therefore lead to an increase in the possibility of missing potential research for inclusion. Similar practical constraints prevented us from basing our focus group sample size on metrics such as code saturation, which may have limited the comprehensiveness of our conclusions. While these are limitations to the presented example of the framework, we believe it is also likely to be representative of the situations many researchers will find themselves in trying to design EMA studies by involving stakeholders.

As EMA research becomes more prevalent, there is great opportunity for progress as researchers learn from the experience and expertise of other investigators and participant stakeholders. Innovation is fundamental to scientific progress, and best practice guidelines assist researches in maintaining intentional, systematic, and replicable procedures while exploring new frontiers. By engaging in the steps outlined herein, researchers conducting EMA studies work collaboratively with stakeholders to increase the relevance and acceptability of their procedures and create a detailed record of their decision-making process, thereby increasing transparency while simultaneously using data to justify their design choices.
